# Evaluation of the risk of hypothyroidism and its clinical manifestations using the Zulewski scale

**DOI:** 10.3389/fendo.2024.1416663

**Published:** 2024-08-16

**Authors:** Aldo Ferreira-Hermosillo, Juan Omar Toledo, Karla Cordoba

**Affiliations:** ^1^ Unidad de Investigación Médica en Enfermedades Endocrinas, Centro Médico Nacional Siglo XXI, Instituto Mexicano del Seguro Social, Mexico City, Mexico; ^2^ Medical Affairs México, MERCK, Naucalpan de Juárez, Mexico

**Keywords:** hypothyroidism, population, Zulewski scale, screening, e-health

## Abstract

**Background:**

Globally, clinical hypothyroidism affects an estimated 0.5 to 5% of the population, while subclinical hypothyroidism affects 5-20%. Limited data is available on the prevalence of thyroid disease within the Mexican population. The objective of this study was to describe the characteristics of people screened for hypothyroidism in Mexico during 2022 using the Zulewski scale.

**Methods:**

A cross-sectional analysis was conducted using data obtained from a digital survey administered by an e-Health platform. This study included participants of all genders, aged 18 years and older (n = 31,449). Descriptive statistics (frequencies and percentages) were sued to describe the data. Differences between groups were assessed through the chi-square or Fischer’s exact test. Information gathered was subjected to hierarchical segmentation analysis to explore trends and patterns. Statistical significance was set as <0.05.

**Results:**

Among the participants, 87.7% were women, and 80% fell within the age group 18 and 44 years. According to the Zulewski scale, 27% of the participants had a low risk of hypothyroidism, 37.4% were classified as having an intermediate risk, and 35.6% were at a high risk. In people at high risk of hypothyroidism, the most common symptom was constipation (29.2%) whereas the most common sign was decreased speed of movement (26.2%). Inquiry of slow movements, dry skin, and facial edema allowed the identification of 90.2% of participants at high risk of hypothyroidism.

**Conclusions:**

In Mexico, a significant portion of the population is at an intermediate or high risk of hypothyroidism, requiring confirmatory diagnostic tests.

## 1 Introduction

Thyroid diseases are among the most prevalent endocrine pathologies. Globally, clinical hypothyroidism affects an estimated 0.5 to 5% of the population, while subclinical hypothyroidism affects 5-20% (1). Its occurrence varies depending on the population under study, influenced by factors such as geographical variations in iodine sufficiency, the age of participants, and the specific different diagnostic thresholds used.

Limited data is available on the prevalence of thyroid disease within the Mexican population (1, 2). In a study conducted by Flores-Rebollar et al. on 427 participants without a previous thyroid disease the results revealed that 1.2% had clinical hypothyroidism (defined as TSH > 4.88 mIU/L and free T4 <0.79 ​​ng/dl), 5.6% had subclinical hypothyroidism (TSH >4.88 mIU/L and free T4 of 0.79 – 1.99 ng/dl) and 8.4% had autoimmune thyroiditis (identified by the presence of anti-peroxidase or anti-thyroglobulin antibodies alongside thyroid hypoechogenicity greater than grade 2) (2). Another study, analyzing data from a cohort of 1750 adult patients aged over 60 years, reported a 7.2% prevalence of clinical hypothyroidism (TSH > 4.8 IU/L and free T4 < 13 pmol/L) and a 15.4% prevalence of subclinical hypothyroidism (TSH > 4.8 IU/L and FT4 13 – 23 pmol/L) (1).

Typically, the clinical manifestations of hypothyroidism are mild and non-specific. Approximately 15% of people with hypothyroidism remain asymptomatic. Others may experience symptoms such as fatigue, lethargy, cold intolerance, weight gain, constipation, dry skin, bradylalia, bradypsychia, and edema, progressing in some cases to the severe condition known as myxedema coma (3). Furthermore, hypothyroidism has been linked to challenges in glycemic control among patients with type 2 diabetes, a higher prevalence of conditions like hypertension, abdominal obesity, dyslipidemia, and cardiovascular disorders (3). It has also shown to be a risk factor for geriatric syndromes including falls, depression, and fatigue (4). Given the substantial variation in the symptomatology of thyroid disorders and the limitation of symptoms alone for accurate detection of hypothyroidism, various screening tools have been developed to identify individuals with previously undiagnosed hypothyroidism and recommended diagnostic testing. The Zulewski scale is the most commonly used instrument for this purpose (5).

The objective of this study was to describe the characteristics of people screened for hypothyroidism in Mexico during 2022 using the Zulewski scale.

## 2 Material and methods

### 2.1 Study design and population

A cross-sectional study was conducted using data obtained from a digital survey conducted by an e-Health platform. The primary objective of the survey was to identify individuals with hypothyroidism or those at high risk of developing it, employing the Zulewski Scale as presented in **Table 1**. This survey was administered as part of a population health campaign spanning various countries in Latin America and the Caribbean taking place between August 5 and August 12, 2022. This report included exclusively data from Mexican participants. The survey was open to individuals of both genders aged 18 years and above. The participants were categorized into following age groups: (i) under 45 years, (ii) 45 to 54 years old, (iii) 55 to 64 years of age and (iv) 65 years and above.

**Table 1:** Questions included in the digital survey.

	Question	Content
	Birthplace	
**Symptoms**	1	What is your sex?
	2	How old are you?
	3	Do you sweat very little, even on hot days?
	4	When you talk or sing, do you have to clear your throat regularly?
	5	Do you have the sensation of tingling or burning without an apparent cause?
	6	Do you suffer from dry skin, or do you have very dry skin?
	7	Do you suffer from constipation or have digestive problems?
	8	Have you lost your hearing little by little?
	9	Have you gained weight for no apparent reason?
**Signs**	10	Have your movements become slow?
	11	Is your reaction speed slow?
	12	Do you feel like the skin on your hands, elbows or forearms is thicker?
	13	Have you noticed any type of swelling on your face?
	14	Do you often feel cold in your hands and feet?

### 2.2 Measurements and variables

The Zulewski Scale serves as a non-invasive screening tool that encompasses signs and symptoms associated with hypothyroidism. The original scale includes symptoms such as reduced sweating (particularly in hot environments or on exceptionally warm days), changes in voice (evident as hoarseness during speech and singing), paresthesia, constipation (including the use of laxatives), progressive hearing impairment, weight gain, and physical signs like as slower of movements, skin thickening (assessed on hands, forearms, elbows) and skin edema (5). According to this scale, a score ranging from 0 to 2 indicates no or a low risk of hypothyroidism (with a negative predictive value [NPV] of 94.2%), 3 to 5 points suggests an intermediate risk while scores above 5 correspond to a to a high risk (with a positive predictive value [PPV] of 96.9%). In the original article, the authors evaluated 50 women with overt hypothyroidism and compared with 80 euthyroid controls and then validated the score in 93 patients with subclinical hypothyroidism. They observed that the most frequent sign was prolonged ankle reflex time (ART, 76%) and the most frequent symptom was dry skin (76%). However, most of the symptoms and signs had PPV higher than 70%, especially slow movements (PPV 96.5%), puffiness (PPV 94.2%), prolonged ART (PPV 92.2%), and loss of hearing (PPV 89.8%). Only pulse rate and cold intolerance had PPV and NPV values below 70%. The values for sensitivity and specificity were 62% and 99% for the cut-off point of 5 (5).

The survey was conducted national scale and was disseminated through press releases and social networks. This approach allowed users to access the platform via the website www.unrecordporlatiroides.com, where they could participate in the survey known as “#Un Record Por La Tiroides”. The website was active only during the specified period and had to meet several requirements: it was managed by a third party responsible for data processing, include terms, conditions as well as privacy policy, could identify the number of surveys completed by the same user, and could produce auditable results.

Upon entering the website, individuals were required to select their country, accept the terms and conditions, and provide their first name, last name, and email address for registration. The user data was protected by data confidentiality agreement. The data processor cleaned the database, removed duplicate entries, and resolved any inconsistencies.

The only inclusion criterium was having a complete questionnaire. Received questionnaires were considered incomplete if they met any of the following criteria: they lacked answers to all the questions, did not progress beyond the initial page of the questionnaire, were abandoned before completion, did not include an acceptance of the terms and conditions or the privacy and cookies policy, or if technical issues causing a loss of connection with the questionnaire were detected. All incomplete questionnaires were subsequently excluded from the analysis.

### 2.3 Statistical analysis

Since the data provided was qualitative, it analyzed using frequencies and percentages. To assess differences between groups, chi-square test or Fisher’s exact test were used taking into consideration the expected and observed numbers in each cell. Additionally, a hierarchical segmentation analysis was conducted. This technique, which is multivariate, explanatory and decompositional employs a sequential, iterative, and descending division process. It begins by defining a discrete dependent variable (variable to be explained) and then forms homogeneous groups based on specific combinations of independent explanatory variables, encompassing all the data collected in the sample. The process is both sequential and interactive, as it examines not only the primary effects of the explanatory variables on the variable being explained but also the interactions between variables. In other words, it explores how each independent variable influences the dependent variable based on of the values ​​taken by other independent variables included in the analysis. Finally, we also evaluated the PPV and the odd ratio (OR) of the signs and symptoms included for detection of hypothyroidism in high risk. The statistical package IBM^®^ SPSS^®^ Statistics Version 26.0 was used for the analysis. A p-value below 0.05 was considered as statistically significant.

## 3 Results

**Figure 1:**
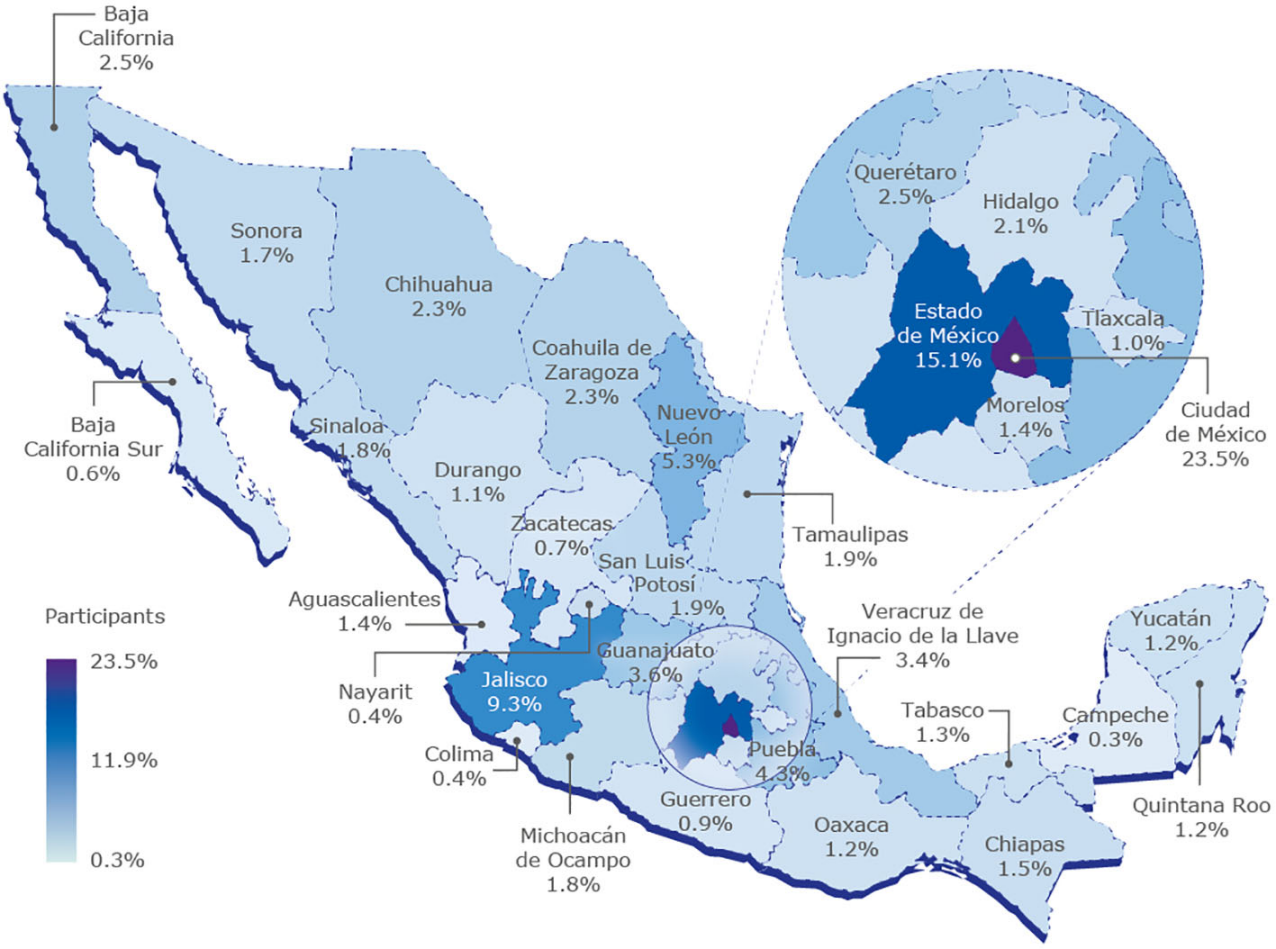
Percentage of study participants according to state.

A total of 31,449 records were obtained and none were excluded as they all met the inclusion criteria (have a complete questionnaire). Among the participants, 87.7% of the participants were women while 12.3% were male. Approximately four out of every five participants fell within the age range of 18 to 44 years. Most study participants were from Mexico City (23.5%), followed by the State of Mexico (15.1%) and Guadalajara (9.3%; **Figure 1**). On the other hand, the states with the lowest participation rates (less than 1%) included Campeche (0.3%), Colima (0.4%), Nayarit (0.4%), Baja California Sur (0.6%), Zacatecas (0.7%) and Guerrero (0.9%).

Among all participants, 60.1% reported experiencing symptoms of constipation, while 47.3% dealt with dry skin and 45.1% reported dysesthesia. Graduate hearing loss was noted by only 21.6% of the respondents. In addition, 41.6% of the participants reported a perception of cold hands and feet, and 38.4% observed a decrease in the speed of movements. According to the Zulewski scale, 27% of the respondents were classified as having no or low risk of hypothyroidism, 37.4% fell into the medium-risk category and 35.6% were classified as high risk.

**Table 2** presents a comparison of the prevalence of symptoms and signs of hypothyroidism according to sex and age group of the study participants. Among women, constipation emerged as the most prevalent symptom, followed by dry skin, weight gain, and dysesthesia. In contrast, hearing loss was the least common symptom, while intolerance to cold was the most frequent sign. For men, none of the symptoms or signs surpassed a 40% prevalence, with frequent throat clearing and slow movements being the most reported symptom and sign. Notably, women more often symptoms associated with hypothyroidism. When assessing the Zulewski scale, 76% of women were categorized as having medium (38%) or high (38%) risk of hypothyroidism, whereas 50% of men were classified as having low or no risk, and 32% fell into the medium-risk category. **Table 3** describes the PPV and the odd ratios (OR) of the signs and symptoms for detecting high risk of hypothyroidism. We observe that constipation, weight gain, dry skin, paresthesias and slow movements had the higher PPVs meanwhile slow movements, slow reaction speed and face swelling had the highest ORs.

**Table 2:** Comparison of the prevalence of symptoms and signs of hypothyroidism according to sex and age group in the study participants.

	Women (n=27581)	Men (n=3868)	*p-value*	≤45 years (n=25,159)	>45 years (n=6290)	*p-value*
Risk						
No/low	24%	50%	*<0.001*	25%	31%	*<0.001*
Mediate	38%	32%		39%	32%	
High	38%	18%		36%	37%	
Symptoms						
Little sweating	40%	30%	*<0.001*	40%	37%	*<0.001*
Need to clear throat	41%	38%	*<0.001*	41%	42%	*<0.001*
Tingling or burning sensation	47%	33%	*<0.001*	45%	44%	*<0.001*
Dry Skin	49%	32%	*<0.001*	47%	48%	*<0.001*
Constipation	64%	34%	*<0.001*	62%	54.5%	*<0.001*
Decreased hearing capacity	22%	20%	*<0.001*	20%	30%	*<0.001*
Weight gain	47%	21%	*<0.001*	45%	41%	*<0.001*
Signs						
Slow movements	40%	28%	*<0.001*	36%	47%	*<0.001*
Slow reaction speed	29%	19%	*<0.001*	27%	31%	*<0.001*
Skin thickening	23%	18%	*<0.001*	23%	20%	*<0.001*
Facial edema	31%	14%	*<0.001*	30%	26%	*<0.001*
Cold intolerance	44%	22%	*<0.001*	44%	33%	*<0.001*

**Table 3:** Diagnostic value of the symptoms and signs for high-risk of hypothyroidism in the study participants.

	PPV	OR (CI95%)
Symptoms		
Little sweating	51.4%	2.2 (2.1 – 2.4)
Need to clear throat	68.9%	6.5 (6.2 – 6.8)
Tingling or burning sensation	75.5%	7.8 (7.4 – 8.2)
Dry Skin	75.1%	6.4 (6.1 – 6.8)
Constipation	82.1%	5.0 (4.7 – 5.3)
Decreased hearing capacity	39.5%	4.9 (4.7 – 5.2)
Weight gain	76.5%	9.4 (8.9 – 9.9)
Signs		
Slow movements	73.5%	11.9 (11.2 – 12.5)
Slow reaction speed	58.4%	11.0 (10.4 – 11.7)
Skin thickening	45.9%	8.1 (7.7 – 8.6)
Facial edema	58.4%	10.1 (9.6 – 10.7)
Cold intolerance	66.6%	5.2 (4.9 – 5.5)

In terms of age, symptoms were statistically significantly more prevalent among participants under 45 years of age in comparison to those aged 45 or older. Among individuals below the age of 45, constipation was the most frequently reported symptom, followed by dry skin, weight gain, and sensations of tingling, tingling, or burning. In this younger age group, 36% were classified as having a high risk of hypothyroidism, 39% were categorized as medium risk and 25% fell into the low-risk category. Among study participants below the age of 45, constipation, dry skin, and sensations of tingling, tingling, or burning were the most prevalent symptoms. Slowness of movement was the most frequently reported sign. Among people over 45 years of age there was the same proportion of individuals classified as having no risk of hypothyroidism (31%) than categorized as medium (32%) or high risk (37%). Thus, in the above 45-year-old participants, two out of three were classified either as being at medium or high risk of hypothyroidism.

Participants under the age of 45 exhibited a higher prevalence of poor sweating, sensations of tingling or pain, constipation, and weight gain. On the other hand, those aged 45 or older had a greater prevalence of frequent throat clearing, dry skin, and decreased hearing ability. In terms of observed signs of hypothyroidism, individuals over 45 of age showed a higher prevalence of slow movements and reduced reaction speed. Conversely, among those under 45 years of age, skin thickening, facial edema and cold intolerance were more commonly reported.

Regardless of the number of participants in each state, more than 50% of the study population was found to be at high risk of hypothyroidism. Notably, in some states such as Mexico City, State of Mexico, Puebla, Tamaulipas, and Veracruz, the prevalence of people at medium risk exceeded those at high risk. Among people classified as at high risk, the most reported symptom was constipation (29.2%) while the least common was decreased hearing ability (14.1%). In this high-risk group, the most prevalent sign in those at high risk was a decrease in the speed of movements (26.2%) whereas the least prevalent sign was thickened skin on the hands, elbows, or forearms (16.4%). Conversely, among individuals at medium risk, the most frequently reported symptom was constipation (23%) with hearing loss being the least common (6.3%). The most common sign for those at medium risk was the sensation of feeling cold (14.8%), while thickened skin on the elbows, hands, or forearms was the least prevalent sign (5.3%).

**Figure 2:**
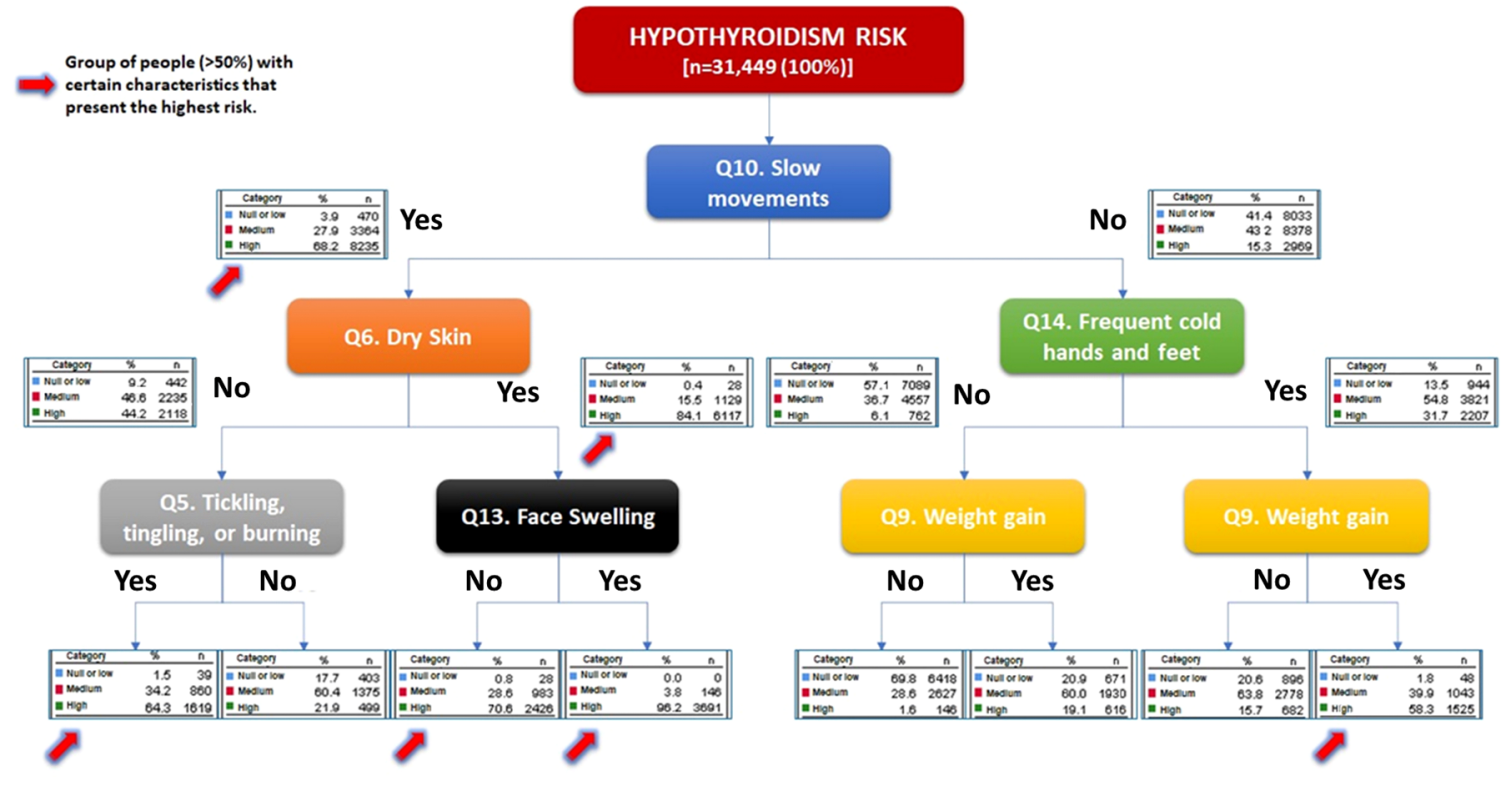
Hierarchical segmentation analysis of symptoms and signs. The presence of slow movements identifies 68% of those patients at high risk of hypothyroidism, if the participant also had dry skin, the possibility increases to 84%; meanwhile the presence of slow movements, dry skin and face swelling allows the identification of 96.2%.

**Figure 2** illustrates the results of a hierarchical segmentation analysis including symptoms and signs of hypothyroidism. The analysis was instrumental in identifying key indicators for categorizing individuals as either high or at low risk of hypothyroidism. The data revealed that the presence of slow movements and dry skin alone could identify approximately 84% of people at high risk of hypothyroidism (p<0.001). In addition, the inclusion information on existence of facial edema increased the probability of hypothyroidism detection to 96.2% (p<0.001). Even in the absence of facial edema, there remained an increased risk of hypothyroidism when both slow movements and dry skin were present. On the other hand, for those without dry skin, inquiring about the presence of tingling or pain increased the probability of identifying individuals at high risk of hypothyroidism by approximately 20% (from 44.2% to 64.3%; p<0.001). Similarly, in study participants without slow movements, the inclusion of questions about cold intolerance increased the probability of identifying high-risk patients from 15.3% to 31.7% (p<0.001). This probability further rose when adding information about weight gain, reaching up to 58.3% (<0.001). The absence of slow movements, cold tolerance or weight gain allowed for the identification of individuals with low or no risk of developing hypothyroidism, with a probability of 69.8% (p<0.001).

## 4 Discussion

The symptoms and signs of hypothyroidism lack specificity when considered in isolation, often resulting in delayed diagnosis and treatment initiation. In fact, some of these symptoms can manifest in people without thyroid disorders. For example, the Danish Investigation of Iodine Intake and Thyroid Diseases study, which involved 140 participants with hypothyroidism and compared them to 560 controls without thyroid disease, revealed that up to 30% of women and 57% of men without thyroid disorders may have more than two symptoms related to hypothyroidism. Notably, the most frequent symptoms included fatigue, shortness of breath, dry skin, emotional lability, palpitations, constipation, and a sensation of a foreign body. Among participants diagnosed with primary hypothyroidism based on laboratory tests, only 13.6% reported experiencing fewer than two related symptoms. However, when a score based on the evaluation of 13 symptoms was calculated, it was observed that the absence of symptoms was more likely to rule out the diagnosis of hypothyroidism in men than in women (6). Considering both sex, the most common symptom was fatigue (86%), followed by dry skin (62%) and shortness of breath (51%), with these symptoms also being most prevalent in women. In our study, constipation was the only symptom present in over 60%, while dry skin and paresthesia were present in less than 50% of the participants. Like the aforementioned study, we found disparities in symptoms and signs between men and women; however, it is important to mention that the number of men in that study was considerably smaller. This could be attributed to the higher risk of hypothyroidism in the women population (7). The fact that constipation was the most frequently reported symptom in our study contrasts with findings from other studies, where fatigue was the most prevalent manifestation, particularly in women (8, 9). For example, a study including 92 Mexican patients with overt hypothyroidism (69.6%) and subclinical hypothyroidism (30.4%), identified fatigue as the most prevalent symptom, which considerably improved after the initiation of levothyroxine (10). The objective of our study was to evaluate the risk of hypothyroidism using a clinical tool among a diverse, large population across Mexico. Thus, we did not investigate the participants’ thyroid profile or other biochemical markers, which would allow us to validate the results obtained by the risk score through laboratory tests. Moreover, in our study did not examine the effect of levothyroxine supplementation on the reported symptoms.

On the other hand, it is worth noting that a higher proportion of participants under the age of 45 participated in our study, and among this group, we identified that 75% had a medium or high risk of hypothyroidism. This higher participation of individuals under 45 coincides with the high prevalence of hypothyroidism typically observed between the ages of 40 and 50 years of age in certain populations (11). For example, a prior study conducted in Taiwan which analyzed data from the Longitudinal Health Insurance Database 2000 included a cohort of one million randomly selected residents from the National Health Insurance data base (12). They reported that out of 18,224 people with thyroid diseases, a total of 22.6% had hypothyroidism. In their study, 47.6% of people with thyroid diseases were between the ages of 18 to 39. Additionally, among patients with hypothyroidism, 78.7% were women, 47.5% were between 40-64 years old and 31% were between 18 and 30 (12). The exact cause of the increased prevalence of hypothyroidism within this age group remains unknown, but in women, it may be linked to hormonal changes during this stage of life. Furthermore, a follow-up study of the Northern Finland Birth Cohort 1966 revealed that the prevalence of hypothyroidism in 340 women experiencing the climacteric phase and 2229 women in the preclimacteric stage at the age of 46 was 5.0%. The study indicated that climacteric was associated with higher prevalence of thyroid dysfunction (OR 1.6, 95% CI 1.1 – 2.3) (13).

In our study, we observed significant differences in the prevalence of all symptoms and signs between the two distinct age groups within our population. A Danish population study comparing young people (under 50 years of age) to old people (over 60 years of age), found differences in the association between symptom presentation and the likelihood of hypothyroidism. Having 0 to 1 symptom was associated with a 25-fold decrease in the chances of hypothyroidism. However, the likelihood of increased substantially when individuals presented with 4 to 8 symptoms, reaching a 4.12-fold increase in risk. Having more than 8 symptoms was associated with a significant 9.17 times higher risk of hypothyroidism. Interestingly, the presence of more than four symptoms proved to be a stronger predictor of hypothyroidism in young people when compared to their older counterparts (OR 16.4, 95% CI 6.69 – 40.0 vs. OR 2.22, 95% CI 1.00 – 4.90) (14). Thus, employing a symptom-based score appeared to be particularly valuable for diagnosing hypothyroidism in younger people. In contrast to our findings, they reported that only dyspnea and wheezing were more prevalent in older adults (14). Our data show that the need to clear the throat, dry skin, and decreased hearing ability, as well as slow movements and slow reaction speed were the most prevalent clinical manifestations in those over 45 years of age (14).

As demonstrated in other studies (14), aggregating various symptoms and signs and creating a composite score has proven to enhance the diagnostic utility for hypothyroidism. In our study, we employed the Zulewski scale, which classified persons with more than 6 points (symptoms/signs) as being at high risk of hypothyroidism. Through hierarchical segmentation analysis, we found that among all the clinical manifestations, inquiring about the presence of slow movements, dry skin, facial edema, and dysesthesia allowed us to identify a significant number of individuals at risk. Therefore, we suggest screening for these manifestations, as an initial step to identify a patient with undiagnosed hypothyroidism.

Naturally, our study has some limitations. It was not possible to measure thyroid hormone levels to validate the risk score with a diagnostic test Although the Zulewski scale has a positive predictive value of 96.9% (5), we recognize that the scale is a prognostic test that requires diagnostic confirmation. Nevertheless, this initial approach aimed to gather more comprehensive data on the national-level risk of hypothyroidism, directly reported by the population. The large number of participants involved in this study enabled the Mexican Society of Nutrition and Endocrinology to achieve a Guinness World Record for the highest number of complete forms. We recognize that some people with undiagnosed overt or subclinical hypothyroidism were more likely to answer the survey. Part of the primary goal of this effort was to increase public awareness regarding the importance of assessing their thyroid profiles and seeking professional medical evaluation if they were identified as being at intermediate or high risk. Undiagnosed hypothyroidism can carry significant clinical implications, particularly for pregnant women (15), older adults (14), and people at increased cardiovascular risk (16–19). Our upcoming next objective will be to compare the thyroid profile of participants based on their risk assessment. This will enable us to validate the diagnostic capacity of the Zulewski scale and assess the correlation between this scale and the number of reported symptoms reported. Another limitation is that our cohort is mainly composed by women. This limited the possibility of assessing differences among the PPV of studied variables or developing a hierarchical segmentation analysis of symptoms and signs by sex. The inclusion of a greater number of women could be related to the 6- to 9-fold increased risk of hypothyroidism in women compared to men, due factors as menopause and aging (6). Furthermore, in Mexico as in some other parts of the world men have less access and use of screening, prevention, and primary care services (20). To our knowledge, no studies have evaluated the utility of the Zulewski scale in men. We encourage that more studies need to be conducted in this population.

In conclusion, according to the Zulewski scale, 73% of the participants in the nationwide thyroid record survey, representing a total of 31,449 individuals from across Mexico were found to be at a medium or high risk of hypothyroidism. Among the symptoms most frequently reported by this diverse population were constipation, dry skin, and dysesthesia, while the most frequent signs included a sensation of cold and a reduced speed of movements. Interestingly, constipation emerged as the most frequently reported symptom, regardless of age and sex. Through a hierarchical segmentation analysis, we observed that asking about the population’s experience of decreased movement speed, dry skin, edema in the hands and feet, and the presence of dysesthesias was particularly insightful in identifying individuals at risk.

## Data Availability

The raw data supporting the conclusions of this article will be made available by the authors, without undue reservation.
