# Corrigendum: Evaluation of the risk of hypothyroidism and its clinical manifestations using the Zulewski scale

**DOI:** 10.3389/fendo.2024.1525162

**Published:** 2025-01-10

**Authors:** Aldo Ferreira-Hermosillo, Juan Omar Toledo, Karla Cordoba

**Affiliations:** ^1^ Unidad de Investigación Médica en Enfermedades Endocrinas, Centro Médico Nacional Siglo XXI, Instituto Mexicano del Seguro Social, Mexico City, Mexico; ^2^ Medical Affairs México, MERCK, Naucalpan de Juárez, Mexico

**Keywords:** hypothyroidism, population, Zulewski scale, screening, e-health

In the published article, there was an error in **Table 2** as published. In the column ≤45 years (n = 25,159), the percentage of “Tingling or burning sensation” and “weight gain” were displayed as “4.5%”. The correct percentage is “45%”. In the column of Men (n = 3868), the percentage of “Constipation” was displayed as “3.4%” and the correct percentage is “34%”. The corrected **Table 2** and its caption Comparison of the prevalence of symptoms and signs of hypothyroidism according to sex and age group in the study participants appear below.

**Table 2:** Comparison of the prevalence of symptoms and signs of hypothyroidism according to sex and age group in the study participants.

	Women (n=27581)	Men (n=3868)	*p-value*	≤45 years (n=25,159)	>45 years (n=6290)	*p-value*
Risk						
No/low	24%	50%	*<0.001*	25%	31%	*<0.001*
Mediate	38%	32%		39%	32%	
High	38%	18%		36%	37%	
Symptoms						
Little sweating	40%	30%	*<0.001*	40%	37%	*<0.001*
Need to clear throat	41%	38%	*<0.001*	41%	42%	*<0.001*
Tingling or burning sensation	47%	33%	*<0.001*	45%	44%	*<0.001*
Dry Skin	49%	32%	*<0.001*	47%	48%	*<0.001*
Constipation	64%	34%	*<0.001*	62%	54.5%	*<0.001*
Decreased hearing capacity	22%	20%	*<0.001*	20%	30%	*<0.001*
Weight gain	47%	21%	*<0.001*	45%	41%	*<0.001*
Signs						
Slow movements	40%	28%	*<0.001*	36%	47%	*<0.001*
Slow reaction speed	29%	19%	*<0.001*	27%	31%	*<0.001*
Skin thickening	23%	18%	*<0.001*	23%	20%	*<0.001*
Facial edema	31%	14%	*<0.001*	30%	26%	*<0.001*
Cold intolerance	44%	22%	*<0.001*	44%	33%	*<0.001*

The authors apologize for this error and state that this does not change the scientific conclusions of the article in any way. The original article has been updated.

